# Lessons learned from the AFLY5 RCT process evaluation: implications for the design of physical activity and nutrition interventions in schools

**DOI:** 10.1186/s12889-015-2293-1

**Published:** 2015-09-23

**Authors:** R. Jago, E. Rawlins, R. R. Kipping, S. Wells, C. Chittleborough, T. J. Peters, J. Mytton, D. A. Lawlor, R. Campbell

**Affiliations:** Centre for Exercise, Nutrition and Health Sciences, School for Policy Studies, University of Bristol, Bristol, BS8 1TZ UK; School of Social and Community Medicine, University of Bristol, Bristol, BS8 2PS UK; School of Population Health, University of Adelaide, Adelaide, SA 5005 Australia; School of Clinical Sciences, University of Bristol, Bristol, BS2 8DZ UK; Public Health, Bristol City Council, Avonquay Office, Cumberland Basin, Bristol, BS1 6XL UK; MRC Integrative Epidemiology Unit at the University of Bristol, Bristol, BS8 2BN UK

**Keywords:** Diet, Physical activity, RCT, Process evaluation, Schools, Children

## Abstract

**Background:**

Systematic reviews have highlighted that school-based diet and physical activity (PA) interventions have had limited effects. This study used qualitative methods to examine how the effectiveness of future primary (elementary) school diet and PA interventions could be improved.

**Methods:**

Data are from the Active For Life Year 5 (AFLY5) study, which was a cluster randomised trial conducted in 60 UK primary schools. Year 5 (8–9 years of age) pupils in the 30 intervention schools received a 12-month intervention. At the end of the intervention period, interviews were conducted with: 28 Year 5 teachers (including 8 teachers from control schools); 10 Headteachers (6 control); 31 parents (15 control). Focus groups were conducted with 70 year 5 pupils (34 control). Topics included how the AFLY5 intervention could have been improved and how school-based diet and PA interventions should optimally be delivered. All interviews and focus groups were transcribed and thematically analysed across participant groups.

**Results:**

Analysis yielded four themes.

Child engagement: Data suggested that programme success is likely to be enhanced if children feel that they have a sense of autonomy over their own behaviour and if the activities are practical.

School: Finding a project champion within the school would enhance intervention effectiveness. Embedding diet and physical activity content across the curriculum and encouraging teachers to role model good diet and physical activity behaviours were seen as important.

Parents and community: Encouraging parents and community members into the school was deemed likely to enhance the connection between schools, families and communities, and “create a buzz” that was likely to enhance behaviour change.

Government/Policy: Data suggested that there was a need to adequately resource health promotion activity in schools and to increase the infrastructure to facilitate diet and physical activity knowledge and practice.

**Discussion and Conclusions:**

Future primary school diet and PA programmes should find ways to increase child engagement in the programme content, identify programme champions, encourage teachers to work as role models, engage parents and embed diet and PA behaviour change across the curriculum. However, this will require adequate funding and cost-effectiveness will need to be established.

**Trial registration:**

ISRCTN50133740

## Background

Low levels of physical activity and fruit and vegetable consumption are associated with increased risk of cardiovascular disease and obesity among adults [1, 2]. Physical activity and fruit and vegetable patterns track from childhood into adulthood [3, 4]. Many children do not consume the recommended five portions of fruit and vegetables per day [5] and do not engage in an hour of moderate to vigorous intensity physical activity (MVPA) [5, 6]. Public health interventions are needed to improve diet and physical activity patterns during childhood and reduce the risk of current and future health problems.

A number of school-based programmes have been evaluated as potential means of changing children’s diet and physical activity behaviours [7–10]. The programmes have been based in schools because the majority of children attend schools, teachers are experts in knowledge provision and behaviour change and there are structures to communicate with children, parents and the wider community [7, 11]. Systematic reviews have reported that the effect of school-based diet, physical activity or obesity prevention programmes have been limited at best [7–9]. We have recently added to this evidence by reporting that there was no difference in accelerometer measured physical activity, sedentary time or self-reported fruit and vegetable consumption among children attending schools who received the curriculum-based Active for Life Year 5 (AFLY5) intervention when compared to a control group about 12 months later [12]. AFLY5 was a large cluster randomized controlled trial that was conducted in 60 primary schools [13, 14]. The intervention included 16 detailed lesson plans and 10 parent–child interactive components and was an adaptation of the Planet Health programme that was shown to have some effect in the US [15]. The content was developed via extensive formative work and refined after a pilot trial [16, 17]. The lessons, delivered by specifically-trained teachers, focussed on increasing physical activity and fruit and vegetable consumption and reducing screen-time and sweetened beverage consumption [13]. The results from this study, taken together with the existing evidence base, indicate a need to identify more effective strategies to improve physical activity and dietary behaviours in primary (elementary) schools.

Evidence from the international health promoting schools literature has suggested that ownership of the health promotion program by school staff, integration of key components into the curriculum, collaboration with key agencies and leadership from school management are likely to be important for school adoption of health promotion programmes [18–20]. It is not clear the extent to which these elements are likely to have affected the implementation and success of the AFLY5 intervention in the UK. These issues can be examined via process evaluations, which can assess intervention fidelity, as well as contextual factors that might explain then intervention and highlight ways in which it could be improved [21–23].

We have recently reported that the intervention was delivered with a high degree of fidelity but some of the intervention materials were perceived by teachers to have become somewhat dated during the time-lag between development and evaluation [24]. Although it would be relatively easy to update intervention materials and modes of delivery, it is also important to identify other factors that could be addressed to enhance the effectiveness of the intervention. The aim of this paper was to use the process evaluation data from AFLY5 to examine the broader lessons learnt to improve the science and art of future diet and physical activity interventions in schools.

## Methods

Data presented here are from the AFLY5 process evaluation, which has been described in detail elsewhere [24, 25]. This paper utilises the parent and teacher interview and child focus group data that were collected at the end of the intervention year to examine responses to the intervention, general views on healthy lifestyle promotion in schools and overall lessons learnt from the project.

The sample for this study included teachers, Headteachers, parents and children. The AFLY5 intervention was delivered by 44 teachers in 30 intervention schools and all 44 intervention teachers were invited to take part in an interview about their experience of AFLY5 with data collected from 20 teachers. The remaining data came from 12 process evaluation schools (six intervention). The process evaluation schools (six intervention) were purposively selected to ensure schools from areas of differing levels of area deprivation and with high or low levels of teaching quality as defined by Office for Standards in Education (Ofsted) Scores (http://www.ofsted.gov.uk/about-us). Schools were invited to participate and recruited into the process evaluation on a first come first served basis. In the intervention schools, interviews were conducted with four Headteachers and 14 parents, with 36 intervention children also participating in six focus groups (ranging from 3 to 8 children per group). In the six control schools interviews were conducted with eight teachers, six Headteachers and 15 parents, and 34 children took part in six focus groups (ranging from 5 to 7 children per group). Pupils who took part in the focus groups were randomly recruited from all children with valid consent to participant in the study in the school. The teacher and Headteacher interviews and focus groups were all conducted in person by ER at the interviewee’s school. The parental interviews were conducted via telephone.

The detailed topic guides for each set of participants are available in the project process evaluation plan [25]. Briefly, however, the topics covered in the teacher interviews included views on health promotion in schools, school involvement in health promotion activities, child engagement in AFLY5 and how the intervention could be improved in the future. The Headteacher interviews assessed the role of health promotion in schools, school involvement in health promotion, thoughts on what makes a successful health promotion strategy in schools, and experiences of participating in AFLY5. Parental interviews asked parents about their thoughts on what is a healthy lifestyle, family diet and physical activity, schools participation in health related activities and their thoughts related to physical activity and nutrition homework activities. The child focus groups asked the children about what they felt they needed to do to be healthy, what lessons they had received in relation to diet and physical activity and what they felt about those lessons and their thoughts on diet and physical activity related homework. All interviews and focus groups were digitally recorded and transcribed verbatim. The mean duration (standard deviation) of the recordings for each group of participants was: 30.6 (10.1) minutes for teachers, 24.3 (7.3) minutes for Headteachers, 36.5 (9.4) minutes for parents and 41.1 (12.5) minutes for students in focus groups.

This study was approved by the Faculty of Medicine and Dentistry Committee for Ethics at the University of Bristol (reference number 111253). All adult participants gave written informed consent, parents gave ‘opt out’ consent for their children and children gave informed assent.

### Analysis

All interviews and focus groups were thematically analysed [26] by ER in NVivo10 (QSR International). Codes were generated both from the topics in the interview guides as well as iteratively from the data. The themes were discussed, refined and agreed by ER, RJ and RC and are illustrated in this paper by selected, anonymised quotes which typify the data. Reporting of the qualitative data is consistent with RATS guidance.

## Results

The themes generated were grouped according to four main categories that reflect the level at which the theme operates: 1) child engagement; 2) school; 3) community level engagement; and 4) Government/Policy (Table 1). These four categories were then broken down further, where necessary, to group together themes into sub-categories relating to various aspects of that level. For example, school level categories are broken down further into organisational, curricular and environmental/ethos related sub categories. In addition, two cross-cutting themes: a) building on existing knowledge and skills; and b) adopting a whole school approach to behaviour change were identified. In the sections below, results are presented by the four levels described above.

**Table 1:** Lessons learnt from AFLY5 & implications for future school-based diet and physical activity (PA) interventions

Lesson learnt	Implication for future research and practice	Cross cutting themes	
1) Child engagement		Building on existing knowledge and resources	Whole school approach
Provide children with a sense of ownership over diet and PA	Create activities that allow children to be actively involved and make choices over behaviours to develop a sense of autonomy		
2) School level			
-Organisational			
Identify a key contact in the school to lead the intervention	Support the teacher to act as a project champion – provide extra training and resource for this person		
-Curricular/ethos			
Embedding diet and PA across the curriculum	Include lessons across the curriculum		
Support teachers to act as positive diet and PA role models	Provide teachers with guidance on how to change their own behaviour and then model key behaviours to reinforce curriculum messages and to improve staff health		
Eating in school	Ensure school meal provision, rules around snacking and packed lunches consistently applied and are supportive of messages in intervention		
Space for physical activity and provision of extra-curricular sports/PA	Identify additional spaces in the school for physical activity		
	Ensure provision of extra-curricular sports/PA consistent/supportive of messages in intervention		
3) Parental and community engagement			
-Parents			
Engage parents with homework	Include activities for parents and children to do together		
Bring the parents into school for events	Have shows, student demonstrations to create a “buzz” around diet and PA behaviour change		
-Wider community			
Invite those with appropriate skills in local area to speak to parents and children	Fits in with the healthy schools framework		
4) Government/policy level			
-Policy/school ethos			
Lack of teacher self-efficacy to teach PA	Increase broader teacher expertise in PA promotion – utilise existing continuing professional development programmes		
Incorporating diet and PA messages across the primary school years	Identify how diet and PA messages are presented across the curriculum and how skills and knowledge are reinforced and developed across the primary school years		
Environmental/setting			
Create facilities for cooking in schools	Negotiated access to school canteens, integrate with school food service. Identify other local resources.		
Create facilities for PA in schools	Identify local resources that could supplement pre-existing school resources.

1. Child engagement

Interviews with teachers and Headteachers indicated that if a project does not involve the children and capture their interest it is not likely to succeed and active lessons in which there are practical components are likely to be more successful. As this Headteacher explained:

*“I think it has to be active, the kids have gotta be feeling like they are involved and that it’s worthwhile, rather than just being talked to.”* (Headteacher, school 56 - Intervention)

*“… it’s got to be fun, practical, so the more the children actually do, rather than just be told*” (Teacher 3, school 56 - Intervention)

One way of ensuring engagement is by creating activities that encourage children to make their own choices over diet and PA behaviours and helping them to develop a sense of autonomy. According to the teachers and Headteachers, in order to enable children to make their own choices they need to understand why it is important to make these changes, rather than just telling them that they should make the changes in order to be healthy. As the following teachers explained, the children need to see the “purpose” behind what they are learning:

*“And so give them some sort of reasoning behind it, why do we eat this and why do we need to exercise and I guess the more they do, they feel it themselves of how, how it affects their bodies”.* (Teacher 3, school 56 - Intervention)

“*I think it’s got, one of these things is the children have got to want to have ownership of it as well and it’s got to be something that they think is again going to be beneficial to them or exciting for them or feel that they are going to make some changes that will happen either for them or for the school*”. (Deputy Headteacher, school 2, control)

2) School level

The school level findings could be captured under two sub-headings, a) organisational and b) curricular/ethos. Each of these issues is discussed below and summarised in Table 1.

- Organisational

The data revealed the importance of having the support of teaching staff in leading and implementing health promotion interventions. Having a key contact to lead the intervention, and having school policies consistent with the intervention, were mentioned by teachers and Headteachers as important aspects of a successful intervention and, in some schools, an element of best practice already present in the school.

*“I think, the school needs to identify somebody who will be given the autonomy to be able to run it and that it is backed up by school policies*.” (Headteacher, school 58 - Intervention)

Having someone in charge championing an intervention was seen to encourage participation.

*“because we’ll do things if somebody organises it and tells us how to do it and plans it and gives us all the bits and pieces to do it with […]”* (Teacher 2, school 3 - Intervention)

*“…we have a lot going on for a small school in terms of sports, which is mainly down to our [Physical Education] PE coordinator who is brilliant*” (Teacher 1, school 32 - Control)

For several participants it was also important for the senior management team to support the project.

*“I’ve seen an awful lot of things go belly up because leadership is very half-hearted and not really involved and has other priorities.”* (Teacher 1, school 54 - Intervention)

b) Curricular and overall ethos

### Teachers as role models

In addition to delivering diet and physical activity related lessons across the curriculum, another important school level theme was to create school policies that ensure teachers acted as role models and adopted the desired behaviours. A number of children commented on their teacher’s dietary habits and more specifically the rumours (or reality) that teachers ate whatever they liked while confiscating similar, “unhealthy” items from the pupils;

*“Well the teachers always tell us off for having chocolate or sweets or anything but it’s really kind of like, they’re kind of not doing that themselves because at break time they always get to have cakes…”* (Focus group, school 56 - Intervention)

*“Yeah, you see a teacher walking past with like a caramel or a Cadbury’s caramel bar or something. It’s so unfair.”* (Focus group, school 56 - Intervention)

It was also clear that the children sometimes saw the rules around what they could eat in school being applied inconsistently, for example, many of the schools had policies around what snacks and items they could bring in for their packed lunch, however, some of the children noticed these were enforced differently within the school. As children in the focus groups explained:

“*They only checked the infants.”* (Focus group, school 32 - Control)

*“I’m not gonna name anyone because I know that it’s private, but there are some people in Year 6 who … their lunch boxes are brimming … they’re going to the brim with things like chocolate and sweets and crisps.”* (Focus group, school 3 - Intervention)

This was also reflected in teacher comments relating to the fact that policies such as lunch box checks, were not always enforced *“as much as they probably should”* (Teacher 2, school 28 - Intervention)

### Embedding diet and PA across the curriculum and school

Many of the school staff highlighted the importance of embedding diet and physical activity programmes across the curriculum. In several interviews it was mentioned that the school was already involved in many smaller projects that would fit with a healthy lifestyles intervention focussing on improving diet and increasing physical activity. For example, a whole school healthy week as this teacher revealed:

“*We do a lot in school because we’re a healthy school, so we do every term there will be, we always focus on the healthy plate and active, active life and the food for life we do, we have a healthy week,”* (Teacher 1, school 32 - Control)

Many of the teachers did not feel confident, or were not interested or engaged, in the delivery of the PA side of the intervention. PE lessons are not always given top priority in primary schools, with some teachers not putting in as much effort to prepare the lessons, despite the fact *“that a good gym lesson is just as carefully prepared as the literacy lesson”* (Headteacher, school 50 - Intervention)

“*We go as far as to talk about drugs and alcohol, smoking, and personally we choose in year five to make it cross-curricula, which we promote across the school anyway, across curricula teaching. But we link into our Tudors topic, so in terms of Henry VIII and his not very healthy diet, and the consequences of that.”*(Teacher 2, school 2 - Control)

In an effort to give the subject specifically trained teachers, there were several schools in the study that had dedicated PE staff to teach their PE lessons; this was seen as a positive step towards good PA provision since

*“it’s someone that actually knows what they’re doing as opposed to teachers that just take PE lessons and get them to run around and do things”* (Teacher 1, school 7 - Control)

Several teachers mentioned that the children in their school had already learnt about healthy lifestyles in earlier school years [grades], and that ideas around good diet and PA practices, therefore, could be introduced earlier in the curriculum, with greater emphasis on personal choice and linking behaviour change to current and future health as the children get older.

*“Well pushing it [AFLY5] throughout the school would probably be great so that they are well aware from Year 1, or from Reception all the way through to Year 5.”* (Teacher 2, school 28 - Intervention)

### Eating in school

Several teachers pointed to the importance of getting parental support for food policies relating to items brought into school in lunchboxes and for break time snacks. Some of the schools already ran projects that involved the whole school, for example a healthy lunchbox initiative that involved children acting as ambassadors for the project;

“*Yeah, well that would be led initially mostly from assemblies, so we’ll start something like that as a whole school initiative, and then there’ll be an assembly where we launch looking at healthy lunch boxes or whatever our healthy, transport or something like that. And then they would, the classes, each individual class will then do a little mini project on it normally and then from there certain children will go on and be ambassadors.”* (Teacher 1, school 2 - Control)

### Space for activity and extra-curricular PA provision

Many schools struggled with timetabling additional time in the hall or gym for physical education sessions. As this teacher highlights with regards to what they feel would be needed to run a successful healthy lifestyles project:

*“[…] we don’t have space and the hall’s time is very limited, so just having the areas. It’s having the space, and having the equipment make it.”* (Teacher 2, school 42 - Intervention)

Lack of equipment and an appropriate space for lessons was also an issue for schools wishing to offer more cookery lessons and practical learning about food and healthy lifestyles. As one of the Headteachers explained:

*“[…] we do a lot about around the science aspect of nutrition, you know will it be nice, it would be nice to do more actual cooking, but again it’s a resource issue”* (Headteacher, school 7 - Control)

While it is not within the remit of health promotion projects, nor are most schools able to reconfigure or build classrooms for cookery lessons, there are options that could involve negotiating access to school canteens, integrating cookery lessons with the school food service or linking with community cooking initiatives, as this school had already explored:

*“But the next thing is just the space you know, to be able to get as many children involved as possible. [****Yeah****]. Our cookery room is about the size of this table”* (Teacher 1, school 2 - Control)

The interviews with Headteachers, teachers and parents revealed that all schools offered extra-curricular physical activities or sports, which were met with varying degrees of success: some were often oversubscribed while others were unable to run due to lack of numbers, as these parents explain:

*“They were trying to get it up and running at school and [child] was really interested and, I sent, − this was back to Christmas now - because I sent back the permission slip and I sent back my money and then had a phone call to say it wasn’t going to happen because there wasn’t enough interest”* (Parent school, 14 - Control)

“*Yeah she is, but er, most of the time she does do after school clubs, it was only this term there must've been a lot of kids trying to do what, ‘cause like she wanted to do tennis, but they couldn’t get in so it's just choir this time.”* (Parent, school 36 - Intervention)

3) Parental and Community engagement

As typified by the quote provided in the eating in school category above, the teachers felt that greater engagement with parents was seen as an important contributor to the success of an intervention. This engagement is important in supporting children at an age when they are not completely independent when it comes to decision making, as this teacher explained:

*“[…] if the children go home all fired up about being healthy but then actually the person doing the shopping doesn’t feel the same, they’re kind of, at this age they’re a bit stuck.”* (Teacher 1, school 2 - Control)

The support of the parents is, therefore, crucial in carrying out healthy lifestyle changes at home since*’ultimately it is the choice of a parent to decide that and to promote that at home”* (Teacher 1, school 45 - Intervention).

*“I think one of the main things, which possibly we don’t do enough of and I would like to promote it more, would be the links to home. So inviting parents in as well to maybe share in cooking lessons.”* (Teacher 2, school 2 - Control)

In order to facilitate greater parental engagement, two main factors were identified as important, homework involving parents and bringing parents into the school for events to create interest around activities. Feedback on parental involvement with the AFLY5 homework activities could be broadly summarised as where parents got on board, they supported AFLY5 on the whole, and in schools where parental involvement was low, parents were not so supportive. It was not always clear why this was the case and for some teachers not receiving any comments from parents was seen as a positive thing, since some parents at their schools would actively complain if there was a problem. Some teachers did receive feedback, which revealed concerns over the intervention being *“a bit preachy”* (Teacher 1, school 56 - Intervention).

It was suggested that bringing parents into school could help to engage them with the project by highlighting the importance of healthy lifestyles and the value of the various elements of the project (such as the homework). Thus, engaging the parents was seen by many as critical and this could be aided by bringing the parents into school to create a buzz around activities.

*“parents are learning alongside their children about healthy eating and what to do and I think for, if you are looking at a long-term thing, I think that’s the type of thing”.* (Headteacher, school 32 - Control)

In addition to providing support for teachers, the impact of having a visitor come in to school was mentioned in several teacher and Headteacher interviews. The excitement of having another person tell children about healthy lifestyles, rather than just hearing from the teacher, was desirable for both child engagement and role modelling:

*“It would be great if you had when you were doing your science lesson on healthy eating, it will be great to have the school nurse or an NHS kind a professional to come in and give some input on that, um, and similar you know, when you’re doing your, your cardiovascular stuff and,’cause the other thing they’ve got, is they’ve got is they’ve got to kit, and children always like to have kit,”* (Headteacher, school 56 - Intervention)

*“Certainly when people, when it’s not the normal teachers, when it’s people coming in from the outside to give a message, that has a huge impact on our children, somebody else speaking, they still listen to what I say but if you’ve got a visitor or somebody that represents something, that often has a bigger impact, definitely.”* (Teacher 3, school 56 - Intervention)

4) Government/Policy

A number of respondents talked about the importance of government support for health promotion initiatives and the need to highlight diet and physical activity as important when compared to other government programmes.

*“And I just think unless you know, that focus becomes on it, because it’s next in line to be focused on, it isn’t gonna happen unfortunately, ‘cause I just think well really all the Government really cares about is whether your child’s achieving national average, not how many times they are having exercise every week or how much they’ve learnt about their bodies.” (Teacher 1a, school 5 - Intervention)*

*“… you know the government says you’re supposed to spend two hours on PE a week, we do try to commit towards that but I wouldn’t say we did that, er with some classes it’s much less than others.”* (Headteacher, school 22 - Control)

### Cross-cutting themes

The sections above have highlighted the four specific themes that were identified within the data. It is, however, also important to acknowledge that these themes can be grouped into two cross-cutting themes: a) building on existing knowledge and resources and; b) adopting a whole school approach which are also presented in Table 1. Specifically, provision of extracurricular programmes, creating spaces and time for physical activity and healthy food activities and inviting external experts into the school are all consistent with building on existing knowledge and resources. Furthermore, embedding diet and physical activity across the curriculum, supporting teachers to act as role models, healthy food procurement and provision, and engaging parents all highlight the importance of adopting a whole school approach.

## Discussion

The data from interviews with teachers and Headteachers presented in this paper indicate that school staff believed that a diet and physical activity focussed health promotion initiative could be effectively delivered in schools, but support or “buy-in” from school staff was critical. This is consistent with previous findings from dietary [27], physical activity [28] and health promoting school [18, 19] intervention literature, and suggests that the development of approaches to secure support from school staff is a crucial phase in the development of any school-based intervention. Consistent with information from the health promoting school field [19], this may be achieved by identifying a key teacher to act as the ‘project champion’ within the school and then providing that staff member with extra training and resources to ensure that the key project messages are delivered in an appropriate manner. If this approach were adopted it would be important to ensure that the project champions fully supported the project and that this was not just a task that was given to the staff member by the school management. Such an approach would be consistent with the Trial for Activity in Adolescent Girls (TAAG) in which a project champion was used to maintain interest in a school-based physical activity intervention once the content sessions were removed [29]. The TAAG analysis showed that the difference between the intervention and control group was enhanced when structured intervention components were removed from the intervention arms and the only difference between the groups was the presence of project champion. This finding offers evidence of the potential importance of a project champion and suggests that identifying project champions from the beginning of a project is likely to be beneficial to the effectiveness of school-based diet and physical activity programmes. Further examination of the ways to support project champions such as through linking with the school nurse or local community health resources, and evidence of ways to motivate potential project champions such as financial reward or alignment with continuing professional development and career progression opportunities are therefore warranted.

The teachers indicated that the impact of the intervention could have been enhanced by greater integration of the content across all years of the curriculum, engaging the teachers as role models and creating a “buzz” around the programme by using external expert speakers. The data also suggested that these changes would be further enhanced by environmental changes such as increased access to cooking facilities and greater extra-curricular physical activity provision and changes to the school ethos to promote healthy eating and physical activity messages across the school. These findings are consistent with the results of previous studies [30, 31] which have highlighted the benefits of engaging parents, and adopting a whole school approach as well as specific studies that have introduced chefs into primary schools [32] and focused on multi-layered interventions [28]. This finding is also consistent with the principal of curriculum integration which has been identified as central to the success of health promoting schools strategies in Scotland [18]. Inchley and colleagues have reported that school staff often feel overwhelmed by constant reforms and that integration within the broader curriculum is essential for the long-term success of health programmes within schools [18]. Thus, curricular changes that would allow diet and physical activity factors to be embedded across the curriculum would mirror the way that some teachers already deliver in depth topics in primary schools.

It is important to highlight that the adoption of a whole school approach for the promotion of a healthy diet and physical activity is possible, but such a change would require considerable integration of curriculum components. It is also important to recognise that our feasibility work with teachers and parents suggested that current intervention was at the limits of a tolerable burden for schools within the current educational structures [17]. Thus, it appears that greater behaviour change is possible via schools but greater recognition at the policy level of the importance of diet and physical activity for child well-being is needed. This could be achieved by increasing the time and content provision for healthy eating and physical activity in the national curriculum. Once recognition is achieved at the governmental level, future work could focus on developing programmes that are much more detailed, integrated and time consuming and therefore also resource intensive. Thus, the challenge for public health is to find ways to garner local and national policy support for fundamental changes to the role that schools play in the development and maintenance of healthy lifestyles among children.

The teachers and pupils suggested that facilitation of greater child autonomy in relation to diet and physical activity choices may enhance the likelihood of intervention success. This would suggest that if the children feel that they are making an informed decision about their current and future health they may be more likely to change their behaviour. AFLY5 was based upon social cognitive theory [33] and a key aim of the programme was to increase the children’s self-efficacy to change their behaviour. It may, therefore be that a greater focus on increasing autonomy, which would be consistent with the central tenants of self-determination theory [34] would assist change in children’s diet and physical activity. Increasingly schools are enabling the voice of the children to be heard through the establishment of student councils and student representatives. Equally, intervention effectiveness is also likely to be enhanced if the teachers feel able and empowered to adapt programme content. These two strategies are consistent with the teacher empowerment and pupil participation strategies that have been identified as important elements of the process by which a school adopts a health promoting school ethos [18]. It may therefore be the case that there is a need for further development work to examine how best to create an environment and ethos in primary schools where teachers feel able to support children to make autonomous decisions about their diet and physical activity and as a result the pupils feel able to change their own behaviour. The increasing evidence base supporting the association between pupil health and educational attainment may help teachers justify inclusion of health promoting components to the curriculum [35].

### Strengths and limitations

The major strength of this paper is the provision of detailed information from parents, children, teachers and Headteachers on how to effectively utilise primary (elementary) schools as a venue to change children’s diet and physical activity behaviours. The results are strengthened by the provision of information from schools that received a physical activity and diet intervention and thus can reflect on content received and by information from control schools who can reflect on current practice. A limitation of the study is that there were a greater number of teachers from intervention than control schools but, in analysing the data, we feel that saturation was reached from all sources. It is also important to recognise that this intervention and the associated process evaluation data were focussed on 8–9 year old children in the UK. During the period of this study there were a number of changes to national policy regarding educational provision which may have influenced the outcomes observed. The UK Government established “Academies” – independent schools, publically funded, but no longer under the control of the local authority. Together with a Government drive towards improving educational attainment and the withdrawal of a mandatory Healthy Schools programme, these may have influenced the motivation and capacity of schools and their staff to engage with the intervention. While it seems likely that the findings from this study will be applicable to schools/institutions with younger and older children, the specifics may differ and therefore a degree of caution is required when relating these findings to other ages and contexts.

## Conclusions

The process evaluation of the Active for Life Year 5 project indicates that changes to the way in which school-based diet and physical activity interventions are delivered would positively affect the success of future diet and physical activity interventions. Future programmes should find ways to increase child engagement in the programme content, identify programme champions, encourage teachers to work as role models, engage parents and embed diet and physical activity behaviour change across the curriculum. Each of these strategies would be consistent with key components of the health promoting schools ethos. As such, our findings may suggest that in addition to curriculum content considerable effort is likely to be needed to develop an environment within schools that can optimise the integration and support of diet and physical activity behaviour change programmes. Specifically, in relation to AFLY5, the data reported in this paper suggest that the intervention effectiveness is likely to have been enhanced by greater integration with the existing curriculum, enhanced support from the school leadership and a broader more extensive school-based programme. It is, however, important to recognise that greater integration of diet and physical activity into the broader curriculum will necessitate fundamental changes to the curriculum which would be most effective if supported by policy makers.

## References

[1] Samitz G, Egger M, Zwahlen M (2011). Domains of physical activity and all-cause mortality: systematic review and dose–response meta-analysis of cohort studies. Int J Epidemiol.

[2] Wang X, Ouyang Y, Liu J, Zhu M, Zhao G, Bao W (2014). Fruit and vegetable consumption and mortality from all causes, cardiovascular disease, and cancer: systematic review and dose–response meta-analysis of prospective cohort studies. BMJ.

[3] Pearson N, Salmon J, Campbell K, Crawford D, Timperio A (2011). Tracking of children’s body-mass index, television viewing and dietary intake over five-years. Prev Med.

[4] Parsons TJ, Power C, Manor O (2006). Longitudinal physical activity and diet patterns in the 1958 British Birth Cohort. Med Sci Sports Exerc.

[5] Hughes RJ, Edwards KL, Clarke GP, Evans CE, Cade JE, Ransley JK (2012). Childhood consumption of fruit and vegetables across England: a study of 2306 6-7-year-olds in 2007. Br J Nutr.

[6] Ness AR, Leary SD, Mattocks C, Blair SN, Reilly JJ, Wells J (2007). Objectively measured physical activity and Fat mass in a large cohort of children. PLoS Med.

[7] Dobbins M, Husson H, DeCorby K, LaRocca RL (2013). School-based physical activity programs for promoting physical activity and fitness in children and adolescents aged 6 to 18. Cochrane Database Syst Rev.

[8] Metcalf B, Henley W, Wilkin T (2012). Effectiveness of intervention on physical activity of children: systematic review and meta-analysis of controlled trials with objectively measured outcomes (EarlyBird 54). BMJ.

[9] Evans CE, Christian MS, Cleghorn CL, Greenwood DC, Cade JE (2012). Systematic review and meta-analysis of school-based interventions to improve daily fruit and vegetable intake in children aged 5 to 12 y. Am J Clin Nutr.

[10] Langford R, Bonell CP, Jones HE, Pouliou T, Murphy SM, Waters E (2014). The WHO Health Promoting School framework for improving the health and well-being of students and their academic achievement. Cochrane Database Syst Rev.

[11] Buse J, Hirst K (2009). The HEALTHY study: introduction. Int J Obes (Lond).

[12] Kipping RR, Howe LD, Jago R, Campbell R, Wells S, Chittleborough CR (2014). Effect of intervention aimed at increasing physical activity, reducing sedentary behaviour, and increasing fruit and vegetable consumption in children: active for Life Year 5 (AFLY5) school based cluster randomised controlled trial. BMJ.

[13] Lawlor DA, Jago R, Noble SM, Chittleborough CR, Campbell R, Mytton J (2011). The Active for Life Year 5 (AFLY5) school based cluster randomised controlled trial: study protocol for a randomized controlled trial. Trials.

[14] Lawlor DA, Peters TJ, Howe LD, Noble SM, Kipping RR, Jago R (2013). The Active for Life Year 5 (AFLY5) school-based cluster randomised controlled trial protocol detailed statistical analysis plan. Trials.

[15] Gortmaker SL, Peterson K, Wiecha J, Sobol AM, Dixit S, Fox MK (1999). Reducing obesity via a school-based interdisciplinary intervention among youth. Arch Pediatr Adolesc Med.

[16] Kipping R, Payne C, Lawlor DA. Randomised controlled trial adapting American school obesity prevention to England. Arch Dis Child 2008. 10.1136/adc.2007.116970 18252756

[17] Kipping RR, Jago R, Lawlor DA (2012). Developing parent involvement in a school-based child obesity prevention intervention: a qualitative study and process evaluation. J Public Health (Oxf).

[18] Inchley J, Muldoon J, Currie C (2007). Becoming a health promoting school: evaluating the process of effective implementation in Scotland. Health Promot Int.

[19] Macnab AJ, Gagnon FA, Stewart D (2014). Health promoting schools: consensus, strategies, and potential. Health Educ.

[20] Deschesnes M, Trudeau F, Kebe M (2010). Factors influencing the adoption of a health promoting school approach in the province of Quebec, Canada. Health Educ Res.

[21] Moore GF, Audrey S, Barker M, Bond L, Bonell C, Hardeman W (2015). Process evaluation of complex interventions: Medical Research Council guidance. BMJ.

[22] Griffin TL, Pallan MJ, Clarke JL, Lancashire ER, Lyon A, Parry JM (2014). Process evaluation design in a cluster randomised controlled childhood obesity prevention trial: the WAVES study. Int J Behav Nutr Phys Act.

[23] Christian MS, Evans CE, Nykjaer C, Hancock N, Cade JE (2014). Evaluation of the impact of a school gardening intervention on children’s fruit and vegetable intake: a randomised controlled trial. Int J Behav Nutr Phys Act.

[24] Rawlins E, Jago R, Wells S, Chittleborough C, Peters TJ, Lawlor DA, Campbell RC. Intervention fidelity in a school-based diet and physical activity intervention in the UK: Active for Life Year 5. Int J Behav Nutr Phys Act In review. 10.1186/s12966-015-0300-7 PMC4642771 26559131

[25] Rawlins E, Campbell R, Kipping RR, Jago R, Wells SL, Lawlor DA. The Active for Life Year 5 (AFLY5) school-based cluster randomised controlled trial protocol: Process Evaluation Plan. School of Social and Community Medicine, University of Bristol: University of Bristol; 2014. http://www.bristol.ac.uk/media-library/sites/social-communitymedicine/migrated/documents/processevaluationplan.pdf.

[26] Grbich C (2007). Qualitative data analysis: an introduction.

[27] Knai C, Pomerleau J, Lock K, McKee M (2006). Getting children to eat more fruit and vegetables: a systematic review. Prev Med.

[28] Gorely T, Nevill ME, Morris JG, Stensel DJ, Nevill A (2009). Effect of a school-based intervention to promote healthy lifestyles in 7–11 year old children. Int J Behav Nutr Phys Act.

[29] Webber LS, Catellier DJ, Lytle LA, Murray DM, Pratt CA, Young DR (2008). Promoting physical activity in middle school girls: trial of activity for adolescent girls. Am J Prev Med.

[30] Christian MS, Evans CE, Ransley JK, Greenwood DC, Thomas JD, Cade JE (2012). Process evaluation of a cluster randomised controlled trial of a school-based fruit and vegetable intervention: Project Tomato. Public Health Nutr.

[31] van Sluijs EM, McMinn AM, Griffin SJ (2007). Effectiveness of interventions to promote physical activity in children and adolescents: systematic review of controlled trials. BMJ.

[32] Caraher M, Seeley A, Wu M, Lloyd S (2013). When chefs adopt a school? An evaluation of a cooking intervention in English primary schools. Appetite.

[33] Bandura A (1986). Social foundations of thought and action: a social cognitive theory.

[34] Deci EL, Ryan RM (2000). The “what” and “why” of goal pursuits: Human needs and the self-determination of behavior. Psychol Inq.

[35] Public Health England. The link between pupil health and wellbeing and attainment: A briefing for head teachers, governors and staff in education settings. Volume PHE publications gateway number: 2014491. Edited by England PH. London: Crown Copyright; 2014;1–14.

